# Supplementary material for: Prevalence of Diabetes and Its Association with Atherosclerotic Cardiovascular Disease Risk in Patients with Familial Hypercholesterolemia: An Analysis from the Hellenic Familial Hypercholesterolemia Registry (HELLAS-FH)
Source: Pharmaceuticals (Basel). 2022 Dec 28;16(1):44. doi: 10.3390/ph16010044 (PMC9863379; doi:10.3390/ph16010044)
Supplement: Supplementary file 1 [file pharmaceuticals-16-00044-s001.zip › pharmaceuticals-2050873-supplementary.pdf]

Supplementary Table S1

Type and dose among statin-treated patients stratified by T2DM status

	T2DM		Non-T2DM		p value (vs. non-T2DM)
	%	Dose (mg/day)	%	Dose (mg/day)	
Atorvastatin	51.7	40 (40-40)	46.1	40 (20-40)	NS
Rosuvastatin	30.0	40 (40-40)	39.6	40 (20-40)	NS
Simvastatin	10.3	40 (25-40)	7.8	40 (20-40)	NS
Pitavastatin	5.7	2 (2-4)	4.7	2 (2-4)	NS
Pravastatin	2.3	40 (40-40)	1.4	40 (20-40)	NS
Fluvastatin	0.0	-	0.4	80 (80-80)	NS

Doses are expressed as mg/day

T2DM: Type 2 diabetes, NS: non-significant.

Supplementary Table S2

Coronary revascularization procedures among the total population stratified by T2DM status

**Parameter (%)**	**T2DM**	**Non-T2DM**	**p value** (vs. non-T2DM)
CABG	14.2	4.5	< 0.05
PCI	23.9	11.5	< 0.05
PCI + CABG	1.6	1.5	NS
None	60.3	82.5	< 0.05

T2DM: Type 2 diabetes, CABG: Coronary Artery Bypass Graft, PCI: Percutaneous Coronary Intervention, NS: non-significant.
